# Awareness of Diabetic Ketoacidosis and Associated Factors Among Adults with Diabetes or a Family Member with Diabetes in Jazan, Saudi Arabia

**DOI:** 10.3390/healthcare14183042

**Published:** 2026-09-16

**Authors:** Omar Oraibi, Saud N. Alwadani, Anwar Darraj, Remaz Abdulaziz Alhassan, Sarah Ali Daghriri, Salman M. Jathmi, Mostafa Mohrag, Luai Alhazmi, Bassem Oraibi, Eman Bahkali, Mohammed Abdulrasak

**Affiliations:** 1 Department of Internal Medicine, College of Medicine, Jazan University, Jazan 45142, Saudi Arabia; ooraibi@jazanu.edu.sa (O.O.); saudwadani78@gmail.com (S.N.A.); anwardraj1423s@gmail.com (A.D.); remazabdulaziz1@gmail.com (R.A.A.); salmanjathmy10@gmail.com (S.M.J.); mmohrag@jazanu.edu.sa (M.M.); lmalhazmi@jazanu.edu.sa (L.A.); 2 King Fahd Central Hospital, Jazan Health Cluster, Jazan 82666, Saudi Arabia; sarahd@moh.gov.sa; 3 Health Research Center, Jazan University, P.O. Box 114, Jazan 82817, Saudi Arabia; boraibi@jazanu.edu.sa; 4 Ministry of National Guard-Health Affairs, Jeddah 22384, Saudi Arabia; bahkaliem@mngah.med.sa; 5 Department of Clinical Sciences, Lund University, 202 13 Malmö, Sweden; 6 Department of Gastroenterology, Skåne University Hospital, 205 02 Malmö, Sweden

**Keywords:** diabetic ketoacidosis, DKA awareness, diabetes mellitus, diabetes education, public awareness, Saudi Arabia, Jazan

## Abstract

**Background**: Diabetic ketoacidosis (DKA) is a life-threatening acute complication of diabetes mellitus that requires prompt recognition and timely management to reduce morbidity and mortality. Public awareness of DKA remains suboptimal in many settings, particularly among individuals with direct exposure to diabetes. This study aimed to assess DKA awareness and identify factors associated with appropriate DKA awareness among adults with personal or familial exposure to diabetes in the Jazan region of Saudi Arabia. **Methods**: A cross-sectional online survey was conducted among adults in the Jazan region between October 2024 and May 2025. Respondents with a personal diagnosis of diabetes or a family member diagnosed with diabetes were included. The questionnaire assessed sociodemographic characteristics, reported diabetes-related characteristics, and DKA awareness across multiple domains, including disease recognition, risk factors, precipitating factors, complications, prevention, and emergency management. Awareness was evaluated using a predefined scoring system, with scores ≥ 6/10 (≥60% of the maximum score) classified as appropriate DKA awareness. Factors associated with appropriate awareness were examined using chi-square tests and multivariable logistic regression. **Results**: A total of 417 respondents were included in the analysis, of whom 150 (36.0%) reported a personal diagnosis of diabetes and 267 (64.0%) reported having a family member with diabetes. Overall, 138 respondents (33.1%; 95% CI, 28.7–37.7%) demonstrated appropriate DKA awareness. Awareness varied across individual domains, with particularly limited recognition of DKA precipitating factors and preventive measures. In multivariable logistic regression analysis, age < 45 years (adjusted odds ratio [aOR] = 3.08, 95% CI: 1.47–6.48; *p* = 0.003), student status (aOR = 2.40, 95% CI: 1.18–4.90; *p* = 0.016), monthly household income > 15,000 SAR (aOR = 4.14, 95% CI: 1.83–9.37; *p* = 0.001), and a personal diagnosis of diabetes (aOR = 2.20, 95% CI: 1.29–3.75; *p* = 0.004) were independently associated with higher odds of appropriate DKA awareness. Reporting type 1 or type 2 diabetes, compared with an unknown diabetes type, was also independently associated with appropriate awareness. Educational attainment was not independently associated with awareness after multivariable adjustment. **Conclusions**: Overall DKA awareness among adults with personal or familial exposure to diabetes in the Jazan region was suboptimal. Appropriate DKA awareness was independently associated with younger age, student status, higher household income, and respondent relationship to diabetes. The association with reported diabetes type should be interpreted cautiously, as it may reflect greater familiarity with diabetes rather than an effect of diabetes subtype itself. These findings support the implementation of targeted educational strategies that include both individuals living with diabetes and their family members to improve recognition of DKA and promote timely healthcare seeking during acute diabetic emergencies. However, the findings should be interpreted in light of the non-probability, social-media-based recruitment strategy, which may have disproportionately represented younger and more highly educated individuals and limits population-level generalizability.

## 1. Introduction

Diabetes mellitus (DM) is a major global public health challenge, affecting an estimated 589 million adults worldwide in 2025, with projections exceeding 850 million by 2050 [1,2]. The burden is particularly high in the Middle East and North Africa (MENA) region, which has the highest comparative prevalence globally [1]. In Saudi Arabia, DM affects approximately 23.1% of adults, making it one of the countries with the highest diabetes prevalence worldwide [3,4,5]. Despite substantial advances in diabetes care, acute complications such as diabetic ketoacidosis (DKA) continue to contribute significantly to morbidity, healthcare utilization, and preventable mortality, underscoring the importance of timely recognition and appropriate management.

Diabetic ketoacidosis (DKA) is one of the most serious acute complications of DM and remains an important cause of emergency department visits, hospital admissions, and diabetes-related mortality despite advances in diabetes management [6,7,8,9,10,11,12]. It results from severe insulin deficiency, leading to hyperglycemia, ketosis, and metabolic acidosis, and commonly presents with polyuria, polydipsia, vomiting, abdominal pain, and altered consciousness [6,7,8,11,12]. Delayed recognition and treatment may result in life-threatening complications, highlighting the importance of early identification by affected individuals, their family members, and healthcare providers [11,12,13,14,15].

Early recognition of DKA is fundamental to improving clinical outcomes because prompt medical assessment and treatment can substantially reduce the risk of severe complications and mortality. Previous studies have shown that inadequate knowledge of DKA warning signs, precipitating factors, sick-day management, and appropriate emergency responses contributes to delayed presentation, recurrent DKA episodes, and avoidable hospitalizations [11,12,13,14,15,16]. Consequently, improving DKA awareness has become an essential component of diabetes education programs, with increasing emphasis on extending education beyond individuals living with diabetes to those who may assist in recognizing acute deterioration and facilitating timely medical care [12,17,18].

Despite increasing recognition of the importance of DKA education and preventive strategies, studies from both high- and low-income countries consistently demonstrate inadequate awareness of DKA symptoms, precipitating factors, preventive measures, and appropriate emergency responses among patients and the general public [19,20,21,22]. Similar findings have been reported across several regions of Saudi Arabia, including Riyadh, Makkah, the Northern and Western regions, and the Northern Borders, highlighting persistent awareness gaps despite ongoing diabetes education initiatives [20,21,22,23]. However, data from the Jazan region remain limited. Furthermore, few studies have evaluated DKA awareness among adults with either a personal diagnosis of diabetes or a diabetic family member, despite both groups being important for the early recognition and timely management of DKA.

Therefore, this study aimed to assess awareness of diabetic ketoacidosis among adults with diabetes or a diabetic family member in the Jazan region of Saudi Arabia and to identify factors associated with appropriate DKA awareness.

## 2. Materials and Methods

### 2.1. Study Design and Setting

A cross-sectional online survey was conducted between October 2024 and May 2025 in the Jazan region of southwestern Saudi Arabia to assess awareness of diabetic ketoacidosis (DKA). Jazan is one of the major administrative regions of the Kingdom of Saudi Arabia, with a population of approximately 1.4 million distributed across urban and rural communities [24]. Similar to the rest of Saudi Arabia, the region has experienced a growing burden of diabetes mellitus (DM), making it an appropriate setting for evaluating public awareness of acute diabetes-related complications [3,4,5].

The study was reported in accordance with the Strengthening the Reporting of Observational Studies in Epidemiology (STROBE) guidelines for cross-sectional studies, and the completed checklist is provided as Supplementary File S1.

### 2.2. Study Population

Adults aged 18 years or older residing in the Jazan region during the study period were eligible to participate. Individuals were included regardless of their diabetes status, provided they either had a personal diagnosis of diabetes mellitus or reported having a family member diagnosed with diabetes. Individuals younger than 18 years of age, non-residents of the Jazan region, and those who declined to provide electronic informed consent were excluded.

Participants were recruited using a non-probability convenience sampling approach through an anonymous online questionnaire disseminated via commonly used social media platforms, including WhatsApp, X (formerly Twitter), and Snapchat. Participation was voluntary, and respondents were informed about the study objectives before providing electronic informed consent. The questionnaire was accessed through an anonymous open link, and no technical restriction was applied to limit individuals to a single submission; therefore, duplicate submissions could not be definitively ruled out. No dedicated automated-bot detection mechanism was implemented. Incomplete questionnaires were excluded, and only completed questionnaires meeting the eligibility criteria were included in the final analysis. Because the survey link was openly disseminated rather than distributed to a defined sampling frame, the number of individuals who received or viewed the survey invitation could not be determined; consequently, a conventional response rate could not be calculated.

The target sample size was estimated using the Raosoft^®^ sample size calculator (Raosoft, Inc., Seattle, WA, USA), assuming a 95% confidence level, a 5% margin of error, and a response distribution of 50%, which yielded a target of at least 385 participants. This calculation was used to guide the recruitment target rather than to imply population representativeness, given the non-probability sampling approach. A total of 417 completed questionnaires were included in the final analysis.

### 2.3. Survey Instrument and Data Collection

Data were collected using a structured, self-administered Arabic-language online questionnaire adapted from the previously published DKA awareness questionnaire developed by Alsaedi et al. [25]. Permission to use and adapt the questionnaire was obtained directly from the original study team. An Arabic version of the original questionnaire was available and used as the basis for the present study; therefore, no additional translation or back-translation was performed. The adapted questionnaire was independently reviewed by two endocrinologists for content relevance, clarity, and appropriateness for the target population. Before the main data collection, the questionnaire was pilot-tested among 20 individuals to assess clarity, comprehensibility, and feasibility. Pilot participants were not included in the final analytical sample. Internal consistency reliability statistics, such as Cronbach’s alpha, were not calculated. Formal psychometric validation, including assessment of construct validity and calculation of a content validity index, was not performed in the present study.

The questionnaire consisted of three sections. The first section collected respondents’ sociodemographic characteristics, including age, sex, nationality, marital status, educational level, employment status, and monthly household income.

The second section collected diabetes-related characteristics according to the respondent’s relationship to diabetes. Respondents with a personal diagnosis of diabetes answered questions regarding their own disease, whereas respondents reporting a family member with diabetes answered the corresponding questions with reference to the affected family member. These questions included diabetes type, duration of diabetes, and previous history of diabetic ketoacidosis. This section also included a separate perception item asking whether patients with diabetes have insufficient knowledge about DKA; this item was descriptive and was not included in the awareness score. Consequently, diabetes-related variables presented in this study refer either to the respondent or to the affected family member, depending on the respondent group.

The third section assessed awareness of diabetic ketoacidosis and was completed by all respondents, irrespective of their relationship to diabetes. The awareness assessment consisted of 10 predefined knowledge items covering DKA recognition, precipitating factors and risk-related knowledge, symptom recognition, risk reduction, complications, and appropriate emergency management. The clinical interpretation of the predefined knowledge items was reviewed against contemporary recommendations for DKA recognition, precipitating factors, prevention, and management [11,12]. The complete questionnaire, including all response options, predefined correct answers, and the detailed scoring method, is provided in Supplementary File S2.

### 2.4. Outcome Measures

The primary outcome was the level of DKA awareness. The awareness score was calculated exclusively from the 10 predefined knowledge items (Q1–Q10) in Section III of the questionnaire. Each item contributed a maximum of one point, yielding a total score ranging from 0 to 10. For the multiple-response symptom-recognition item (Q9), one point was awarded when all five predefined correct symptoms were identified; otherwise, no point was awarded. The separate perception item regarding whether patients with diabetes have insufficient knowledge about DKA was descriptive and was not included in the awareness score.

Consistent with the classification approach used in the source study [25], participants achieving ≥60% of the maximum awareness score were classified as having appropriate DKA awareness. Accordingly, participants with a total score of ≥6/10 were categorized as having appropriate awareness, whereas those scoring <6/10 were categorized as having inappropriate awareness. This threshold was adopted from the classification approach of the source study and was not derived from receiver operating characteristic analysis or formal psychometric validation in the present study. The complete questionnaire, predefined correct answers, and detailed scoring method are provided in Supplementary File S1.

### 2.5. Statistical Analysis

Data were analyzed using the Statistical Package for the Social Sciences (SPSS), version 27.0 (IBM Corp., Armonk, NY, USA). Categorical variables were summarized as frequencies and percentages, whereas continuous variables were summarized using means and standard deviations, as appropriate.

Associations between respondent characteristics and DKA awareness were initially evaluated using the Chi-square test or Fisher’s exact test, where appropriate. Binary logistic regression was subsequently used to identify factors associated with appropriate DKA awareness. The dependent variable was awareness level, categorized as appropriate (score ≥ 6/10) or inappropriate (score < 6/10). Crude odds ratios (cORs) with 95% confidence intervals (CIs) were estimated using univariable logistic regression. The multivariable logistic regression model included sex, age, educational level, employment status, monthly household income, respondent relationship to diabetes (personal diagnosis versus family member), diabetes type, previous history of DKA, and duration of diabetes. For regression analyses, age was dichotomized as <45 vs. ≥45 years, and educational level was collapsed as secondary education or higher versus below secondary education to reduce sparse-category effects, limit the number of model parameters, and improve model stability. The original categories were retained in the descriptive and bivariate analyses. These variables were selected a priori because they represented the principal sociodemographic and diabetes-related characteristics collected in the study and were considered potentially relevant to DKA awareness; variable inclusion was not based solely on statistical significance in the univariable analyses. Adjusted odds ratios (aORs) with 95% CIs were reported.

Only complete eligible questionnaires were included in the analytical dataset, and no missing-data imputation was required. Multicollinearity among the explanatory variables was assessed using variance inflation factors (VIFs), which ranged from 1.15 to 2.85, indicating no important multicollinearity. Model calibration was evaluated using the Hosmer–Lemeshow goodness-of-fit test, which showed no evidence of poor fit (χ^2^ = 10.38, df = 8, *p* = 0.239).

As a sensitivity analysis, associations involving reported diabetes type, previous DKA history, and diabetes duration were additionally examined after stratifying respondents according to their relationship to diabetes (personal diagnosis versus reporting a diabetic family member). Exact tests were used where appropriate because of sparse cell counts.

Statistical significance was defined as a two-sided *p*-value < 0.05.

For interpretation of the regression analyses, diabetes-related clinical variables, including diabetes type, duration of diabetes, and previous history of DKA, referred either to the respondent’s own clinical characteristics or to those of the affected family member, depending on the respondent’s relationship to diabetes. This distinction was maintained throughout the analyses and interpretation of the study findings.

## 3. Results

### 3.1. Baseline Characteristics

A total of 417 respondents were included in the analysis. The baseline sociodemographic and reported diabetes-related characteristics of the study respondents are summarized in Table 1. Among the respondents, 150 (36.0%) reported a personal diagnosis of diabetes, whereas 267 (64.0%) reported having a family member diagnosed with diabetes. The study population was almost equally distributed by sex, with most respondents aged 18–24 years and holding a university degree. Approximately half reported a monthly household income below SAR 5000. Regarding the reported diabetes-related characteristics, Type 1 and Type 2 diabetes were reported at similar frequencies, approximately one-third reported a previous history of diabetic ketoacidosis, whereas most reported a diabetes duration exceeding five years.

### 3.2. DKA Awareness

Participants demonstrated varying levels of awareness regarding diabetic ketoacidosis (DKA) (Table 2). Nearly half (48.2%) correctly identified DKA as a medical emergency, whereas 31.2% were uncertain of its definition. Approximately three-fifths recognized that DKA is life-threatening and may affect multiple organs. However, awareness of precipitating factors was limited, with only 39.3% identifying missed insulin doses as a major precipitating factor and approximately one-third responding correctly to the questionnaire items concerning infection and physical exertion. Less than half correctly responded to the item concerning glycemic control (normal HbA1c) and DKA risk reduction, while 16.8% identified all five predefined correct symptoms in the multiple-response symptom-recognition item. Overall, 55.6% correctly identified immediate hospital referral as the appropriate response to suspected DKA. Significant differences between males and females were observed for the definition of DKA (*p* = 0.026), the item concerning missed insulin doses as a precipitating factor (*p* = 0.027), and the symptom-recognition item (*p* = 0.031).

**Figure 1:**
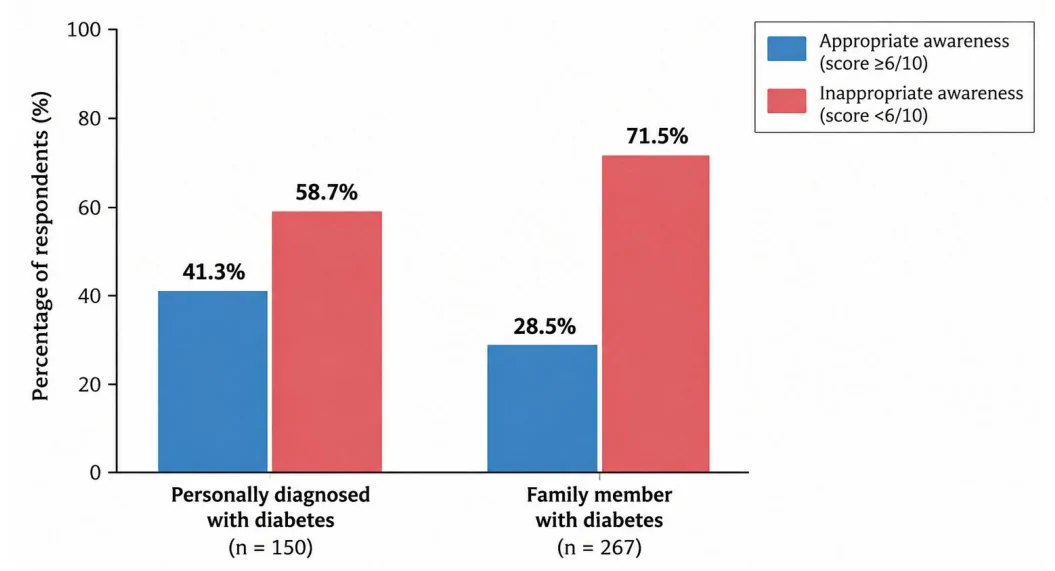
Distribution of DKA awareness level according to respondents’ relationship to diabetes. Appropriate DKA awareness was defined as a total awareness score ≥ 6/10 (≥60% of the maximum score).

Overall, 138 of the 417 respondents (33.1%) demonstrated appropriate DKA awareness (95% CI, 28.7–37.7%), whereas 279 (66.9%) were classified as having inappropriate awareness (Figure 1 and Table 3). Appropriate DKA awareness was significantly associated with age (*p* = 0.002), educational level (*p* = 0.031), monthly household income (*p* = 0.001), employment status (*p* = 0.007), respondent relationship to diabetes (*p* = 0.009), and reported diabetes type (*p* < 0.001). In contrast, no significant associations were observed for sex, marital status, reported previous history of DKA, or reported duration of diabetes (all *p* > 0.05).

Respondents with a personal diagnosis of diabetes demonstrated a significantly higher proportion of appropriate DKA awareness than respondents reporting a diabetic family member (41.3% vs. 28.5%, *p* = 0.009). Appropriate awareness also differed significantly according to reported diabetes type, with substantially lower awareness among respondents who reported that the diabetes type was unknown. This finding should be interpreted cautiously, as knowledge of the reported diabetes type may reflect greater familiarity with diabetes rather than a true difference in DKA awareness according to diabetes subtype.

### 3.3. Multivariable Analysis

Multivariable logistic regression analysis identified several factors independently associated with appropriate DKA awareness (Table 4). Age < 45 years was associated with higher odds of appropriate awareness compared with age ≥ 45 years (aOR = 3.08, 95% CI: 1.47–6.48; *p* = 0.003). Educational level was not independently associated with appropriate awareness after adjustment (aOR = 1.47, 95% CI: 0.48–4.50; *p* = 0.503). Students had significantly greater odds of appropriate awareness than unemployed respondents (aOR = 2.40, 95% CI: 1.18–4.90; *p* = 0.016), whereas the association for employed respondents was not statistically significant after adjustment.

Respondents with a monthly household income exceeding SAR 15,000 had higher odds of appropriate DKA awareness than those with a monthly income below SAR 5000 (aOR = 4.14, 95% CI: 1.83–9.37; *p* = 0.001). In addition, respondents with a personal diagnosis of diabetes had higher odds of appropriate DKA awareness than respondents reporting a family member with diabetes (aOR = 2.20, 95% CI: 1.29–3.75; *p* = 0.004).

Participants who reported type 1 diabetes (aOR = 4.41, 95% CI: 1.94–10.04; *p* < 0.001) or type 2 diabetes (aOR = 5.50, 95% CI: 2.52–11.98; *p* < 0.001) had higher odds of appropriate DKA awareness compared with participants who reported that the diabetes type was unknown. Previous history of DKA and duration of diabetes were not independently associated with appropriate DKA awareness.

Given the potential heterogeneity between self-reported and proxy-reported clinical information, an additional sensitivity analysis was performed after stratifying respondents according to their relationship to diabetes (Supplementary Table S1). Among respondents personally diagnosed with diabetes (*n* = 150), appropriate DKA awareness was significantly more frequent among those reporting a previous history of DKA than among those without previous DKA (55.4% vs. 27.6%; *p* = 0.001). In contrast, among respondents reporting a diabetic family member (*n* = 267), appropriate awareness was lower when previous DKA was reported for the affected family member (17.5% vs. 31.4%; *p* = 0.047). Reported diabetes type was significantly associated with awareness in both strata (*p* = 0.003 and *p* < 0.001, respectively), whereas diabetes duration was not significantly associated with awareness in either stratum (*p* = 0.268 and *p* = 0.759, respectively). These findings suggest heterogeneity between self-reported and proxy-reported clinical characteristics and support cautious interpretation of the pooled clinical-variable estimates.

## 4. Discussion

This study evaluated diabetic ketoacidosis (DKA) awareness among adults with personal or familial exposure to diabetes in the Jazan region of Saudi Arabia. Overall, only 33.1% of respondents demonstrated appropriate DKA awareness, highlighting substantial awareness gaps despite direct exposure to diabetes. Appropriate DKA awareness was independently associated with younger age, higher household income, student status, a personal diagnosis of diabetes, and reporting a known diabetes type. These findings suggest that although direct exposure to diabetes may improve awareness, substantial gaps in recognizing and understanding DKA persist, emphasizing the need for targeted educational interventions for both individuals living with diabetes and their families.

The present study found that only 33.1% of respondents demonstrated appropriate DKA awareness, indicating that overall awareness of this potentially life-threatening complication remains suboptimal despite participants having either a personal diagnosis of diabetes or a family member affected by the disease. This finding is consistent with previous studies conducted in Saudi Arabia and other countries, which have similarly reported inadequate awareness among adults with personal or familial exposure to diabetes and persistent misconceptions regarding DKA symptoms, precipitating factors, and emergency management [19,20,21,22,23,25,26,27]. The relatively low awareness observed in our study may reflect limited access to structured diabetes education, insufficient emphasis on acute diabetes complications during routine healthcare encounters, and reliance on informal sources of health information [17,18,19,20,21,22,23,28,29]. Given that delayed recognition of DKA may contribute to delayed healthcare seeking and worse clinical outcomes, these findings underscore the need for comprehensive educational initiatives targeting not only individuals living with diabetes but also their family members, who often play a critical role in recognizing symptoms and facilitating timely medical care [12,17,18].

Age, employment status, and household income were independently associated with appropriate DKA awareness in the present study. Respondents aged <45 years had significantly higher odds of appropriate awareness than those aged ≥ 45 years. Students had significantly greater odds of appropriate awareness than unemployed respondents, while respondents with a monthly household income exceeding SAR 15,000 also had significantly higher odds of appropriate awareness than those with a monthly income below SAR 5000. Although higher educational attainment was associated with appropriate awareness in the univariable analysis, this association did not remain statistically significant after multivariable adjustment. These findings are consistent with previous studies reporting that higher educational attainment and socioeconomic status are associated with improved diabetes-related knowledge and health literacy [18,20,21,22,23,28,29]. Individuals with higher levels of education may have better access to health information, greater ability to understand medical advice, and enhanced engagement with educational resources. Likewise, students and respondents with higher incomes may have greater access to educational resources, health information, and healthcare services, which may contribute to improved awareness of diabetes complications. However, these factors were not directly assessed in the present study and therefore should be interpreted with caution. These findings highlight the importance of developing educational interventions that are tailored to individuals with lower educational attainment and limited socioeconomic resources, thereby reducing disparities in DKA awareness across different population groups.

Respondents with a personal diagnosis of diabetes demonstrated significantly greater DKA awareness than those reporting a diabetic family member. This finding is biologically and clinically plausible, as individuals living with diabetes are more likely to receive structured education regarding acute diabetes complications during routine clinical care, diabetes education programs, and follow-up visits. In contrast, although family members frequently play an important role in supporting diabetes self-management and recognizing medical emergencies, they may have fewer opportunities to receive formal education about DKA unless specifically included in diabetes education initiatives. These findings highlight the importance of extending diabetes education beyond individuals living with diabetes to include family members, as improving awareness among household members may facilitate earlier recognition of DKA symptoms, timely healthcare seeking, and potentially better clinical outcomes during acute diabetic emergencies [17,18,25].

Participants who reported type 1 or type 2 diabetes had higher odds of appropriate DKA awareness than those who reported that the diabetes type was unknown. This finding should be interpreted cautiously. Rather than indicating that diabetes type itself is associated with greater DKA awareness, the observed association may reflect greater familiarity with diabetes among respondents who knew the specific diabetes type, whether their own or that of an affected family member. Knowledge of diabetes type may therefore serve as a marker of greater engagement with, or understanding of, diabetes-related health information. Accordingly, these findings should not be interpreted as evidence of a true difference in DKA awareness attributable to diabetes subtype itself.

The stratified sensitivity analysis further suggested heterogeneity between the two respondent groups, particularly for previous DKA history, for which the direction of association with awareness differed between respondents personally diagnosed with diabetes and family-member respondents. This finding reinforces the need for caution when interpreting pooled associations involving clinical characteristics derived from a combination of self-reported and proxy-reported information.

In contrast, sex, reported previous history of DKA, and reported duration of diabetes were not independently associated with appropriate DKA awareness after multivariable adjustment. Age, however, was independently associated with awareness in the revised analysis, with respondents aged <45 years demonstrating higher odds of appropriate awareness than those aged ≥45 years. Similarly, the absence of an independent association between reported previous DKA history and awareness may indicate that experiencing DKA does not necessarily translate into sustained knowledge, particularly if structured education and reinforcement are limited following the acute event. Likewise, the duration of diabetes was not independently associated with appropriate DKA awareness, indicating that longer disease duration was not associated with greater awareness in this study. These findings emphasize that ongoing diabetes education and regular reinforcement of key messages may be more important than disease duration or previous clinical experience in improving DKA awareness [10,17,18].

This study has several strengths. To our knowledge, it is among the few studies in Saudi Arabia to comprehensively evaluate DKA awareness among adults with personal or familial exposure to diabetes, including both individuals with a personal diagnosis of diabetes and those reporting a diabetic family member. The questionnaire assessed multiple domains of DKA-related knowledge, including disease recognition, risk factors, precipitating factors, complications, prevention, and emergency management, providing a broad evaluation of respondents’ knowledge related to DKA. Furthermore, the use of multivariable logistic regression enabled adjustment for potential confounding factors and facilitated the identification of factors independently associated with appropriate DKA awareness. Collectively, these strengths provide valuable insights into awareness gaps and may help inform the development of targeted educational strategies aimed at improving early recognition and management of DKA within the community.

This study has several limitations that should be considered when interpreting the findings. First, its cross-sectional design precludes causal inference between respondent characteristics and DKA awareness. Second, data were collected using an online, self-administered questionnaire and may therefore be subject to recall and social desirability biases. In addition, participants were recruited through convenience sampling using an anonymous open survey link distributed via social media platforms. This approach may have introduced selection bias and overrepresented individuals with greater digital access, interest in diabetes, or health awareness, including younger and more educated participants. Because probability-based sampling was not used, the study sample should not be considered representative of the adult population of Jazan, and the observed level of DKA awareness should not be interpreted as a population-representative estimate. Furthermore, because the survey was distributed through an anonymous open link without a technical restriction limiting individuals to a single submission, duplicate responses could not be definitively excluded. The number of individuals who received or viewed the survey invitation was also unknown; therefore, a conventional response rate could not be calculated.

Third, diabetes-related clinical information was reported differently according to the respondent group. Participants with diabetes reported their own clinical characteristics, whereas participants without diabetes provided proxy information regarding an affected family member. Proxy-reported information regarding variables such as diabetes type, disease duration, and previous DKA may be less accurate than information reported directly by individuals with diabetes, potentially introducing information bias, misclassification, and additional heterogeneity. Although respondent relationship to diabetes was explicitly considered in the analyses and included in the multivariable model, residual differences between the two respondent groups may remain. Accordingly, associations involving diabetes-related clinical characteristics should be interpreted with caution. Furthermore, the association between reported diabetes type and DKA awareness may partly reflect respondents’ knowledge of the affected individual’s diabetes type rather than a true difference in awareness according to diabetes type. In addition, some items included in the awareness score, particularly those concerning physical exertion and HbA1c, assess risk-factor or risk-reduction knowledge rather than direct recognition of DKA and may therefore have limited face validity as measures of overall DKA awareness. Accordingly, the composite awareness score should be interpreted as a measure of broader DKA-related knowledge rather than a direct measure of DKA recognition alone. Although the questionnaire underwent expert review and pilot testing, formal psychometric validation, including calculation of a content validity index and internal consistency measures such as Cronbach’s alpha, was not performed in the present study; therefore, the measurement properties of the composite awareness score should be interpreted with caution. Finally, the study was conducted in a single region of Saudi Arabia, which further limits the generalizability of the findings to populations with different demographic and healthcare characteristics. Despite these limitations, the study provides evidence regarding gaps in DKA awareness among adults with personal or familial exposure to diabetes and identifies groups that may benefit from targeted educational interventions.

## 5. Conclusions

This study demonstrated that overall awareness of diabetic ketoacidosis (DKA) among adults with personal or familial exposure to diabetes in the Jazan region was suboptimal, with only 33.1% of respondents demonstrating appropriate DKA awareness. Appropriate DKA awareness was independently associated with younger age, student status, higher household income, and a personal diagnosis of diabetes. Reporting a known diabetes type was also associated with appropriate awareness; however, this finding may reflect greater familiarity with diabetes rather than an effect of diabetes subtype itself. These findings highlight the need for targeted educational strategies that extend beyond individuals living with diabetes to include family members, with particular emphasis on improving recognition of DKA symptoms, precipitating factors, and appropriate emergency management. Future studies should evaluate the effectiveness of structured educational interventions in improving DKA awareness and their impact on timely recognition and healthcare-seeking behavior during acute diabetic emergencies.

## Figures and Tables

**Table 1:** Sociodemographic characteristics of the respondents and reported diabetes-related characteristics † (*N* = 417).

Variable	*n* (%)
**Sociodemographic characteristics**	
**Age group (years)**	
18–24	176 (42.2)
25–34	88 (21.1)
35–44	62 (14.9)
45–54	53 (12.7)
55–64	22 (5.3)
≥65	16 (3.8)
**Sex**	
Male	207 (49.6)
Female	210 (50.4)
**Educational level**	
No formal education	24 (5.8)
Primary	9 (2.2)
Intermediate	12 (2.9)
Secondary	100 (24.0)
University	264 (63.3)
Postgraduate	8 (1.9)
**Marital status**	
Single	209 (50.1)
Married	190 (45.6)
Divorced	11 (2.6)
Widowed	7 (1.7)
**Monthly household income (SAR)**	
<5000	209 (50.1)
5000–10,000	90 (21.6)
10,000–15,000	78 (18.7)
>15,000	40 (9.6)
**Employment status**	
Student	147 (35.3)
Employed	162 (38.8)
Unemployed	108 (25.9)
**Respondent relationship to diabetes**	
Respondent diagnosed with diabetes	150 (36.0)
Respondent reporting a diabetic family member	267 (64.0)
**Reported diabetes-related characteristics †**	
Type 1 diabetes	170 (40.8)
Type 2 diabetes	168 (40.3)
Unknown	79 (18.9)
**Reported previous history of diabetic ketoacidosis**	
Yes	131 (31.4)
No	286 (68.6)
**Reported duration of diabetes**	
1–5 years	114 (27.3)
6–10 years	140 (33.6)
>10 years	163 (39.1)

Abbreviations: DKA, diabetic ketoacidosis. **†** Reported diabetes-related characteristics were provided by the respondent and refer either to the respondent’s own diabetes (respondents diagnosed with diabetes) or to the diabetes history of the affected family member (respondents reporting a diabetic family member), according to the questionnaire design.

**Table 2:** Responses to diabetic ketoacidosis (DKA) awareness questions according to sex among the study respondents (*n* = 417).

DKA Awareness Question	Response	Female *n* (%)	Male *n* (%)	Overall *n* (%)	*p*-Value
**DKA is best described as:**					**0.026**
	A medical emergency resulting from diabetes complications requiring urgent treatment	100 (47.6)	101 (48.8)	201 (48.2)	
	A chronic complication of diabetes that occurs over time and does not require urgent treatment	20 (9.5)	38 (18.4)	58 (13.9)	
	A natural physiological response to diabetes	14 (6.7)	14 (6.8)	28 (6.7)	
	I don’t know	76 (36.2)	54 (26.1)	130 (31.2)	
**DKA is a life-threatening condition:**					**0.443**
	Yes	124 (59.0)	132 (63.8)	256 (61.4)	
	No	11 (5.2)	13 (6.3)	24 (5.8)	
	I don’t know	75 (35.7)	62 (30.0)	137 (32.9)	
**DKA may affect other organs, including the brain and heart:**					**0.443**
	Yes	107 (51.0)	117 (56.5)	224 (53.7)	
	No	18 (8.6)	13 (6.3)	31 (7.4)	
	I don’t know	85 (40.5)	77 (37.2)	162 (38.8)	
**DKA affects children only:**					**0.357**
	Yes	47 (22.4)	59 (28.5)	106 (25.4)	
	No	66 (31.4)	60 (29.0)	126 (30.2)	
	I don’t know	97 (46.2)	88 (42.5)	185 (44.4)	
**Which of the following is the most common precipitating cause of DKA?**					**0.027**
	Forgetting to take insulin	68 (32.4)	96 (46.4)	164 (39.3)	
	Irregular eating and exercise habits	33 (15.7)	26 (12.6)	59 (14.1)	
	Having diabetes for a long period	24 (11.4)	23 (11.1)	47 (11.3)	
	I don’t know	85 (40.5)	62 (30.0)	147 (35.3)	
**Infection can be a cause of DKA:**					**0.083**
	Yes	54 (25.7)	74 (35.7)	128 (30.7)	
	No	44 (21.0)	39 (18.8)	83 (19.9)	
	I don’t know	112 (53.3)	94 (45.4)	206 (49.4)	
**Knowledge item regarding physical exertion as a potential DKA precipitating factor:**					**0.145**
	Yes	62 (29.5)	70 (33.8)	132 (31.7)	
	No	35 (16.7)	45 (21.7)	80 (19.2)	
	I don’t know	113 (53.8)	92 (44.4)	205 (49.2)	
**Knowledge item regarding glycemic control (normal HbA1c) and DKA risk reduction:**					**0.534**
	Yes	95 (45.2)	93 (44.9)	188 (45.1)	
	No	15 (7.1)	21 (10.1)	36 (8.6)	
	I don’t know	100 (47.6)	93 (44.9)	193 (46.3)	
**Identification of all five predefined correct DKA symptoms (multiple-response item):**					**0.031**
	**All five predefined correct symptoms identified**	**27 (12.9)**	**43 (20.8)**	**70 (16.8)**	
	**All five predefined correct symptoms not identified**	**183 (87.1)**	**164 (79.2)**	**347 (83.2)**	
**Appropriate initial management of suspected DKA:**					**0.262**
	Give the patient water and wait for improvement	8 (3.8)	14 (6.8)	22 (5.3)	
	Give the patient sugar orally and wait for improvement	28 (13.3)	36 (17.4)	64 (15.3)	
	Call an ambulance and transfer the patient immediately to the hospital	119 (56.7)	113 (54.6)	232 (55.6)	
	I don’t know	55 (26.2)	44 (21.3)	99 (23.7)	
**Participant perception (not included in the awareness score)**					
**In your opinion, do diabetic patients have low awareness of DKA?**					**0.266**
	Yes	46 (21.9)	55 (26.6)	101 (24.2)	
	No	164 (78.1)	152 (73.4)	316 (75.8)	

Abbreviations: DKA, diabetic ketoacidosis; HbA1c, glycated hemoglobin. Note: *p* values were calculated using the Chi-square test. The DKA awareness score was calculated exclusively from the 10 predefined knowledge items (Q1–Q10). For the multiple-response symptom-recognition item (Q9), a correct response required identification of all five predefined correct symptoms, as detailed in Supplementary File S2. The participant-perception item regarding whether patients with diabetes have insufficient knowledge about DKA was descriptive and was not included in the awareness score. The wording of these items reflects the questionnaire administered to participants; the complete questionnaire and predefined scoring key are provided in Supplementary File S2.

**Table 3:** Association between respondents’ characteristics and appropriate diabetic ketoacidosis (DKA) awareness (*n* = 417).

Variable	Inappropriate DKA Awareness *n* (%)	Appropriate DKA Awareness *n* (%)	*p*-Value
**Sex**			
Male	136 (65.7)	71 (34.3)	0.677
Female	143 (68.1)	67 (31.9)	
**Age (years)**			**0.002**
18–24	106 (60.2)	70 (39.8)	
25–34	55 (62.5)	33 (37.5)	
35–44	43 (69.4)	19 (30.6)	
45–54	39 (73.6)	14 (26.4)	
55–64	21 (95.5)	1 (4.5)	
≥65	15 (93.8)	1 (6.3)	
**Educational level**			**0.031** *
No formal education	23 (95.8)	1 (4.2)	
Primary	7 (77.8)	2 (22.2)	
Intermediate	9 (75.0)	3 (25.0)	
Secondary	65 (65.0)	35 (35.0)	
University	171 (64.8)	93 (35.2)	
Postgraduate	4 (50.0)	4 (50.0)	
**Marital status**			0.086 *
Single	132 (63.2)	77 (36.8)	
Married	135 (71.1)	55 (28.9)	
Divorced	6 (54.5)	5 (45.5)	
Widowed	6 (85.7)	1 (14.3)	
**Monthly household income (SAR)**			**0.001**
<5000	139 (66.5)	70 (33.5)	
5000–10,000	71 (78.9)	19 (21.1)	
10,000–15,000	52 (66.7)	26 (33.3)	
>15,000	17 (42.5)	23 (57.5)	
**Employment status**			**0.007**
Student	86 (58.5)	61 (41.5)	
Employed	110 (67.9)	52 (32.1)	
Unemployed	83 (76.9)	25 (23.1)	
**Respondent relationship to diabetes**			**0.009**
Respondent diagnosed with diabetes	88 (58.7)	62 (41.3)	
Respondent reporting a diabetic family member	191 (71.5)	76 (28.5)	
**Reported diabetes type †**			**<0.001**
Type 1 diabetes	105 (61.8)	65 (38.2)	
Type 2 diabetes	104 (61.9)	64 (38.1)	
Unknown	70 (88.6)	9 (11.4)	
**Reported previous history of diabetic ketoacidosis †**			0.108
Yes	81 (61.8)	50 (38.2)	
No	198 (69.2)	88 (30.8)	
**Reported duration of diabetes †**			0.647
1–5 years	78 (68.4)	36 (31.6)	
6–10 years	96 (68.6)	44 (31.4)	
>10 years	105 (64.4)	58 (35.6)	
**Overall DKA awareness level**	**279 (66.9)**	**138 (33.1)**	
**95% CI for appropriate DKA awareness**		**28.7–37.7%**	

Abbreviations: DKA, diabetic ketoacidosis; CI, confidence interval. Notes: *p* values were calculated using the Chi-square test. * Fisher’s exact test was used where appropriate. **†** Reported diabetes-related variables refer either to the respondent’s own diabetes (respondents diagnosed with diabetes) or to the diabetes history of the affected family member (respondents reporting a family member with diabetes).

**Table 4:** Factors associated with appropriate diabetic ketoacidosis (DKA) awareness among the study respondents: univariable and multivariable logistic regression analyses (*n* = 417).

Variable	Crude OR (95% CI)	*p*-Value	Adjusted OR (95% CI)	*p*-Value
**Sex**				
Male *	1	—	1	—
Female	0.90 (0.60–1.35)	0.603	1.23 (0.76–1.99)	0.397
**Age**				
<45 years	2.18 (1.25–3.79)	**0.006**	3.08 (1.47–6.48)	**0.003**
≥45 years *	1	—	1	—
**Educational level**				
Less than secondary *	1	—	1	—
Secondary or above	3.58 (1.47–8.67)	**0.005**	1.47 (0.48–4.50)	0.503
**Employment status**				
Student	2.36 (1.33–4.18)	**0.003**	2.40 (1.18–4.90)	**0.016**
Employed	1.57 (0.91–2.72)	0.107	1.67 (0.74–3.76)	0.214
Unemployed *	1	—	1	—
**Monthly household income (SAR)**				
<5000 *	1	—	1	—
5000–10,000	0.53 (0.29–0.95)	**0.033**	0.77 (0.39–1.50)	0.435
10,000–15,000	0.99 (0.57–1.71)	0.964	1.57 (0.86–2.87)	0.141
>15,000	2.69 (1.36–5.32)	**0.005**	4.14 (1.83–9.37)	**0.001**
**Respondent relationship to diabetes**				
Respondent diagnosed with diabetes	1.77 (1.16–2.69)	**0.008**	2.20 (1.29–3.75)	**0.004**
Respondent reporting a diabetic family member *	1	—	1	—
**Reported diabetes type †**				
Type 1 diabetes	4.81 (2.26–10.23)	**<0.001**	4.41 (1.94–10.04)	**<0.001**
Type 2 diabetes	4.79 (2.25–10.21)	**<0.001**	5.50 (2.52–11.98)	**<0.001**
Unknown *	1	—	1	—
**Reported previous history of DKA †**				
Yes	1.46 (0.95–2.25)	0.087	1.13 (0.66–1.93)	0.668
No *	1	—	1	—
**Reported duration of diabetes †**				
1–5 years *	1	—	1	—
6–10 years	0.99 (0.57–1.72)	0.977	0.73 (0.41–1.31)	0.295
>10 years	1.20 (0.69–2.07)	0.515	0.70 (0.40–1.23)	0.216

Abbreviations: OR, odds ratio; CI, confidence interval; DKA, diabetic ketoacidosis; cOR, crude odds ratio; aOR, adjusted odds ratio. Notes: * Reference category. † Reported diabetes-related variables refer either to the respondent’s own diabetes (respondents diagnosed with diabetes) or to the diabetes history of the affected family member (respondents reporting a family member with diabetes). Age and educational level were collapsed for multivariable regression to reduce sparse-category effects and improve model stability; original categories are presented in Table 3.

## Data Availability

The data supporting the findings of this study are available from the corresponding author upon reasonable request. The data are not publicly available due to privacy considerations related to participant survey data.

## Supplementary Materials

The following supporting information can be downloaded at https://www.mdpi.com/article/10.3390/healthcare14183042/s1, Supplementary File S1: STROBE Statement—Completed Checklist for Cross-Sectional Study. Supplementary File S2: DKA Awareness Questionnaire and Scoring Key. Supplementary Table S1: Stratified Sensitivity Analysis of DKA Awareness According to Respondent Relationship to Diabetes.
