# Supplementary material for: Awareness of Diabetic Ketoacidosis and Associated Factors Among Adults with Diabetes or a Family Member with Diabetes in Jazan, Saudi Arabia
Source: Healthcare (Basel). 2026 Sep 16;14(18):3042. doi: 10.3390/healthcare14183042 (PMC13606367; doi:10.3390/healthcare14183042)
Supplement: Supplementary file 1 [file healthcare-14-03042-s001.zip › Supplementary File S1 – STROBE Checklist.pdf]

STROBE Statement - Completed Checklist for Cross-Sectional Study

Manuscript: Awareness of Diabetic Ketoacidosis and Associated Factors Among Adults with Diabetes or a Family Member with Diabetes in Jazan, Saudi Arabia

Item No.	Recommendation	Page No.	Relevant text from manuscript
**Title and abstract**			
1(a)	Indicate the study design with a commonly used term in the title or the abstract.	2	Abstract Methods identifies a cross-sectional online survey.
1(b)	Provide in the abstract an informative and balanced summary of what was done and what was found.	2-3	Abstract summarizes background, methods, sample (N=417), overall awareness with 95% CI, multivariable results, and conclusions.
**Introduction**			
2	Explain the scientific background and rationale for the investigation being reported.	3-4	Introduction describes the burden and clinical importance of DKA, prior awareness gaps, and the rationale for evaluating adults with personal or familial exposure to diabetes.
3	State specific objectives, including any prespecified hypotheses.	4	The final Introduction paragraph states the objective: assess DKA awareness and identify factors associated with appropriate awareness in Jazan.
**Methods**			
4	Present key elements of study design early in the paper.	4	Section 2.1 identifies a cross-sectional online survey.
5	Describe the setting, locations, and relevant dates, including periods of recruitment, exposure, follow-up, and data collection.	4	Jazan region, Saudi Arabia; data collection from October 2024 to May 2025.
6(a)	Cross-sectional study: Give the eligibility criteria, and the sources and methods of selection of participants.	4-5	Section 2.2 provides eligibility criteria and describes convenience recruitment through an anonymous online questionnaire disseminated via WhatsApp, X, and Snapchat.
6(b)	Matching criteria / numbers for matched cohort or case-control studies.	N/A	Not applicable to this unmatched cross-sectional study.
7	Clearly define all outcomes, exposures, predictors, potential confounders, and effect modifiers. Give diagnostic criteria, if applicable.	5-7	Sections 2.3-2.5 define the DKA awareness outcome, scoring threshold, sociodemographic and diabetes-related variables, and covariates included in regression.
8*	For each variable of interest, give sources of data and details of methods of assessment. Describe comparability of assessment methods if there is more than one group.	5-6	Questionnaire source, respondent-specific reporting of clinical variables, expert review/pilot testing, and awareness scoring are described. Self- versus proxy-reported clinical information is explicitly distinguished.
9	Describe any efforts to address potential sources of bias.	4-5, 19-20	Methods describe open-link recruitment and survey-quality limitations; Limitations address selection bias, recall/social-desirability bias, duplicate submissions, proxy reporting, and measurement limitations.
10	Explain how the study size was arrived at.	5	Target sample size estimated with Raosoft using 95% confidence, 5% margin of error, and 50% response distribution; target at least 385 participants.
11	Explain how quantitative variables were handled in the analyses. If applicable, describe which groupings were chosen and why.	6-7	Section 2.5 describes summaries and prespecified collapsing of age (<45 vs. >=45 years) and education for regression to reduce sparse-category effects and improve model stability.
12(a)	Describe all statistical methods, including those used to control for confounding.	6-7	Chi-square/Fisher exact tests and univariable/multivariable logistic regression are described; cORs/aORs with 95% CIs are reported. Multicollinearity and model calibration were assessed.
12(b)	Describe any methods used to examine subgroups and interactions.	7, 15-16	Sensitivity analysis stratified respondents by personal diabetes versus reporting a diabetic family member; exact tests were used because of sparse cells.
12(c)	Explain how missing data were addressed.	7	Only complete eligible questionnaires were included; no missing-data imputation was required.
12(d)	Cross-sectional study: If applicable, describe analytical methods taking account of sampling strategy.	4-5	Convenience/social-media sampling is described. Probability-based sampling and survey weighting were not used; population representativeness is not claimed.
12(e)	Describe any sensitivity analyses.	7, 15-16	Stratified sensitivity analysis examined diabetes type, previous DKA history, and diabetes duration according to respondent relationship to diabetes.
**Results**			
13(a)	Report numbers of individuals at each stage of study (e.g., potentially eligible, examined, confirmed eligible, included, and analysed).	5, 7	A total of 417 completed eligible questionnaires were included and analyzed; incomplete questionnaires were excluded.
13(b)	Give reasons for non-participation at each stage.	4-5	Eligibility/exclusion criteria and exclusion of incomplete questionnaires are stated. Because the link was openly disseminated, the number viewing the invitation and a conventional response rate could not be determined.
13(c)	Consider use of a flow diagram.	N/A	No participant flow diagram was used; recruitment involved a single cross-sectional online survey.
14(a)	Give characteristics of study participants and information on exposures and potential confounders.	7-9	Table 1 reports sociodemographic and reported diabetes-related characteristics of all 417 respondents.
14(b)	Indicate number of participants with missing data for each variable of interest.	7	No missing-data imputation was required because only complete eligible questionnaires were analyzed.
14(c)	Cohort study: Summarise follow-up time.	N/A	Not applicable; this was a cross-sectional study.
15*	Cross-sectional study: Report numbers of outcome events or summary measures.	12-15	Overall appropriate DKA awareness was 138/417 (33.1%; 95% CI 28.7%-37.7%); Table 3 reports awareness according to respondent characteristics.

Item No.	Recommendation	Page No.	Relevant text from manuscript
16(a)	Give unadjusted estimates and, if applicable, confounder-adjusted estimates and their precision. Make clear which confounders were adjusted for and why they were included.	15-17	Table 4 reports crude and adjusted ORs with 95% CIs and p-values. Section 2.5 states the variables included in the multivariable model and that they were selected a priori.
16(b)	Report category boundaries when continuous variables were categorized.	6-7, 14-17	Category boundaries/reference categories are reported for age, education, household income, and diabetes duration.
16(c)	If relevant, consider translating estimates of relative risk into absolute risk for a meaningful time period.	N/A	Not applicable; the study reports odds ratios for a cross-sectional awareness outcome.
17	Report other analyses done, e.g., analyses of subgroups and interactions, and sensitivity analyses.	15-16	The stratified sensitivity analysis is reported by respondent relationship to diabetes, including exact p-values for diabetes type, prior DKA, and diabetes duration.
**Discussion**			
18	Summarise key results with reference to study objectives.	17-19	Discussion summarizes overall awareness and the principal factors independently associated with appropriate DKA awareness.
19	Discuss limitations of the study, taking into account sources of potential bias or imprecision. Discuss both direction and magnitude of any potential bias.	19-20	Limitations address cross-sectional design, recall/social-desirability and digital selection bias, duplicate submissions/bot detection, proxy reporting, measurement validity, and single-region scope; implications for interpretation are stated.
20	Give a cautious overall interpretation of results considering objectives, limitations, multiplicity of analyses, results from similar studies, and other relevant evidence.	17-20	Discussion compares findings with prior studies and repeatedly cautions against causal or subtype-specific interpretation, including pooled self/proxy clinical variables.
21	Discuss the generalisability (external validity) of the study results.	19-20	The manuscript states that convenience social-media recruitment and conduct in a single Saudi region limit population-level generalizability and that the sample should not be considered representative of Jazan adults.
**Other information**			
22	Give the source of funding and the role of the funders for the present study and, if applicable, for the original study on which the present article is based.	20	Funding: This research received no external funding.

*Information is reported separately by respondent relationship to diabetes where relevant. N/A = not applicable.
