# Supplementary material for: Awareness of Diabetic Ketoacidosis and Associated Factors Among Adults with Diabetes or a Family Member with Diabetes in Jazan, Saudi Arabia
Source: Healthcare (Basel). 2026 Sep 16;14(18):3042. doi: 10.3390/healthcare14183042 (PMC13606367; doi:10.3390/healthcare14183042)
Supplement: Supplementary file 1 [file healthcare-14-03042-s001.zip › Supplementary File S2 DKA Questionnaire.pdf]

Supplementary File S2

DKA Awareness Questionnaire and Scoring Key

Awareness of Diabetic Ketoacidosis and Associated Factors Among Adults with Diabetes or a Family Member with Diabetes in Jazan, Saudi Arabia

Supplementary note. The questionnaire was administered in Arabic using Google Forms. The English text below is a faithful translation and organized presentation of the questionnaire used in the study. The original wording and response options of the administered questionnaire have been retained. Scoring annotations, identification of correct responses, and the scoring key are provided retrospectively for transparency and were not displayed to participants.

Participant Information and Consent

We are a group of researchers from the College of Medicine at Jazan University conducting a research study to assess awareness of diabetic ketoacidosis among adults with diabetes or a family member with diabetes in Jazan, Saudi Arabia.

Completion of this questionnaire indicates your consent to participate in this research. All information collected will be kept strictly confidential and will be used for research purposes only.

Thank you for your cooperation.

Eligibility and consent: Are you 18 years of age or older and do you agree to participate in this questionnaire?

○ Yes

○ No

Section SI. Sociodemographic Characteristics

1. Age (years):

○ 18–24

○ 25–34

○ 35–44

○ 45–54

○ 55–64

○ 65 and above

2. Sex:

○ Male

○ Female

3. Nationality:

○ Saudi

○ Non-Saudi

4. Educational level:

○ No formal education

○ Primary

○ Intermediate

○ Secondary

○ University

○ Postgraduate

5. Marital status:
○ Single
○ Married
○ Divorced
○ Widowed
6. Monthly household income (SAR):
○ <5,000
○ 5,000–10,000
○ 10,000–15,000
○ >15,000
7. Employment status:
○ Student
○ Employed
○ Unemployed

Section SII. Diabetes-Related Characteristics
8. What is your relationship to diabetes?
○ I have been diagnosed with diabetes mellitus
○ A family member has diabetes mellitus
○ Neither / No
9. What is the type of diabetes mellitus?
○ Type 1 diabetes
○ Type 2 diabetes
○ I do not know
10. Has the person with diabetes previously been diagnosed with diabetic ketoacidosis (DKA)?
○ Yes
○ No
11. What is the duration of diabetes?
○ 1–5 years
○ 6–10 years
○ More than 10 years
12. In your opinion, do patients with diabetes have insufficient knowledge about diabetic ketoacidosis (DKA)?
○ Yes
○ No
Important scoring note: Question 12 is a descriptive perception/opinion item and was not included in the DKA awareness score.

Section SIII. Assessment of Awareness of Diabetic Ketoacidosis (DKA)

Scoring note for this supplementary research copy: Correct responses are identified below in bold. These markings were not shown to participants.

Q1. Diabetic ketoacidosis (DKA) is regarded as:

○ **An emergency event that occurs as a complication of diabetes and requires urgent treatment**

○ A chronic complication of diabetes that occurs over a long period and does not require urgent treatment

○ A normal physiological change that occurs in response to diabetes

○ I do not know

Q2. Diabetic ketoacidosis (DKA) is considered a life-threatening condition.

○ **Yes**

○ No

○ I do not know

Q3. Diabetic ketoacidosis (DKA) may affect other systems in the body, such as the brain and heart.

○ **Yes**

○ No

○ I do not know

Q4. Diabetic ketoacidosis (DKA) affects children only.

○ Yes

○ **No**

○ I do not know

Q5. One of the main causes of diabetic ketoacidosis (DKA) is:

○ **Missing an insulin dose**

○ Irregular eating and exercise

○ Having diabetes for a long period

○ I do not know

Q6. Infection can be one of the causes of diabetic ketoacidosis (DKA).

○ **Yes**

○ No

○ I do not know

Q7. Physical exertion can be one of the causes of diabetic ketoacidosis (DKA).

○ **Yes**

○ No

○ I do not know

Q8. The incidence of diabetic ketoacidosis (DKA) decreases when a normal HbA1c level is maintained.

○ **Yes**

○ No

○ I do not know

Q9. Which of the following symptoms may indicate that a patient with diabetes has diabetic ketoacidosis (DKA)? (Select all that apply.)

☐ Fatigue and tiredness

☐ **Loss of consciousness**

☐ **Nausea**

☐ **Abdominal pain**

☐ **Rapid or difficult breathing**

☐ **Characteristic or unusual breath odor**

Q9 scoring rule: Q9 was treated as one awareness item. One point was awarded when all five predefined correct symptoms (loss of consciousness, nausea, abdominal pain, rapid or difficult breathing, and characteristic/unusual breath odor) were selected. Selection of fatigue/tiredness did not negate the point. Otherwise, Q9 received 0 points.

Q10. What is the appropriate urgent action for a patient with diabetes who is experiencing diabetic ketoacidosis (DKA)?

○ Give the patient water and wait until the patient improves

○ Give the patient oral sugar and wait until the patient improves

○ **Call an ambulance and take the patient to the hospital immediately**

○ I do not know

Scoring Key for the 10 DKA Awareness Items

The awareness score was calculated exclusively from Q1–Q10 in Section SIII. Each item contributed a maximum of one point, yielding a total score from 0 to 10. The separate perception item in Section SII was not scored.

Item	Correct response / scoring criterion	Points
Q1	DKA is an emergency complication requiring urgent treatment.	1
Q2	Yes — DKA is life-threatening.	1
Q3	Yes — DKA may affect the brain and heart.	1
Q4	No — DKA does not affect children only.	1
Q5	Missing an insulin dose.	1
Q6	Yes — infection can precipitate DKA.	1
Q7	Yes, as predefined in the source questionnaire and original scoring scheme.*	1
Q8	Yes, as predefined in the source questionnaire and original scoring scheme.*	1
Q9	All five predefined correct symptoms identified; fatigue/tiredness does not negate the point.	1
Q10	Call an ambulance and take the patient to the hospital immediately.	1

**Q7 and Q8 were retained and scored according to the predefined scoring of the administered/source questionnaire. Physical exertion is not considered a major established precipitating factor for DKA in contemporary consensus guidance, and the HbA1c item reflects risk-related or risk-reduction knowledge rather than direct DKA recognition. These items therefore have limited face validity as direct measures of DKA awareness.*

Overall Awareness Classification

Total possible score: 0–10 points.

Appropriate DKA awareness: ≥6/10 (≥60% of the maximum score).

Inappropriate DKA awareness: <6/10 (<60% of the maximum score).

The ≥60% classification criterion follows the percentage-based classification approach used in the source study from which the questionnaire was adapted (Alsaedi et al., 2024; Cureus 16:e60336; doi:10.7759/cureus.60336).
