# Supplementary material for: Awareness of Diabetic Ketoacidosis and Associated Factors Among Adults with Diabetes or a Family Member with Diabetes in Jazan, Saudi Arabia
Source: Healthcare (Basel). 2026 Sep 16;14(18):3042. doi: 10.3390/healthcare14183042 (PMC13606367; doi:10.3390/healthcare14183042)
Supplement: Supplementary file 1 [file healthcare-14-03042-s001.zip › Supplementary Table S1.pdf]

Supplementary Table S1. Stratified Sensitivity Analysis of DKA Awareness According to Respondent Relationship to Diabetes

Clinical characteristic	Personally diagnosed, n=150	p	Family-member respondents, n=267	p
**Diabetes type**	T1: 46/91 (50.5%); T2: 16/51 (31.4%); Unknown: 0/8 (0%)	**0.003**	T1: 21/79 (26.6%); T2: 46/117 (39.3%); Unknown: 9/71 (12.7%)	**<0.001**
**Previous DKA**	Yes: 41/74 (55.4%); No: 21/76 (27.6%)	**0.001**	Yes: 10/57 (17.5%); No: 66/210 (31.4%)	**0.047**
**Diabetes duration**	1–5 y: 14/38 (36.8%); 6–10 y: 20/56 (35.7%); >10 y: 28/56 (50.0%)	0.268	1–5 y: 22/76 (28.9%); 6–10 y: 26/84 (31.0%); >10 y: 28/107 (26.2%)	0.759
